# Regulation of Huntingtin Gene Expression by miRNA-137, -214, -148a, and Their Respective isomiRs

**DOI:** 10.3390/ijms140816999

**Published:** 2013-08-19

**Authors:** Emilia Kozlowska, Wlodzimierz J. Krzyzosiak, Edyta Koscianska

**Affiliations:** Department of Molecular Biomedicine, Institute of Bioorganic Chemistry, Polish Academy of Sciences, Noskowskiego 12/14 Str., 61-704 Poznan, Poland; E-Mail: emiliak@ibch.poznan.pl

**Keywords:** miRNA, isomiR, target validation, luciferase assay, huntingtin, Huntington’s disease

## Abstract

With the advent of deep sequencing technology, a variety of miRNA length and sequence variants, termed isomiRNAs (isomiRs), have been discovered. However, the functional roles of these commonly detected isomiRs remain unknown. In this paper, we demonstrated that miRNAs regulate the expression of the *HTT* gene, whose mutation leads to Huntington’s disease (HD), a hereditary degenerative disorder. Specifically, we validated the interactions of canonical miRNAs, miR-137, miR-214, and miR-148a, with the HTT 3′UTR using a luciferase assay. Moreover, we applied synthetic miRNA mimics to examine whether a slight shifting of miRNA seed regions might alter the regulation of the *HTT* transcript. We also examined miR-137, miR-214, and miR-148a isomiRs and showed the activity of these isoforms on reporter constructs bearing appropriate sequences from the HTT 3′UTR. Hence, we demonstrated that certain 5′-end variants of miRNAs might be functional for the regulation of the same targets as canonical miRNAs.

## 1. Introduction

MicroRNAs (miRNAs) are 21- to 24-nucleotide noncoding RNAs that fine-tune gene expression. These molecules act at the posttranscriptional level through modulation of translational efficiency and/or destabilization of target transcripts (reviewed in [1]). miRNAs exert their functions through imperfect pairing with the 3′ untranslated region (UTR) of target mRNAs. Nucleotides 2 through 8 of the miRNA, termed the “seed” sequence, are essential for target recognition and binding [2].

The canonical pathway of animal miRNA biogenesis includes two subsequent cleavages (reviewed in [3–6]). Briefly, precursor miRNAs (~60-nt pre-miRNAs) are generated from primary transcripts (pri-miRNAs) through cleavage with the ribonuclease Drosha and exported to the cytoplasm by Exportin-5. Then, ~22-nt miRNA duplexes are generated through cleavage with the ribonuclease Dicer. Only one miRNA strand (the guide strand) of the duplex induces Argonaute proteins (AGO) to form the programmed RNA-induced silencing complex (RISC); the other strand (the passenger strand, or miRNA*) is released and degraded. The thermodynamic stability of the ends of the miRNA duplexes plays a crucial role in miRNA strand selection.

Currently, more than 2000 mature human miRNAs have been deposited in the miRNA repository (miRBase, Release 19) [7]. The deep sequencing of short RNAs has not only enabled the identification of novel miRNAs but also revealed that miRNAs are heterogeneous and differ in length. Heterogeneous miRNA variants are referred to as isomiRNAs (isomiRs) [8]. The primary source of the heterogeneity of miRNA length is imprecise cleavage by the ribonucleases Drosha and Dicer [8–11], which can be further biased at the AGO2 binding step [12]. However, miRNA length variation might also reflect various downstream effects, such as limited miRNA degradation by exonucleases, the addition of extra nucleotides [13–15], and miRNA sequencing artifacts [16,17]. It has recently been shown that the human trans-activation response (TAR) RNA-binding protein (TRBP), a molecular partner of Dicer, might also contribute to miRNA length heterogeneity. Specifically, TRBP triggered the production of isomiRs that were longer at the 5′ strand than the canonical miRNAs by a single nucleotide. As a result, different mRNAs were targeted due to changes in guide-strand selection [18]. It has also been reported in *Drosophila* that the Nibbler (Nbr) 3′–5′ exonuclease trims the 3′ ends of miR-34 generating isomiRs shorter than the canonical sequence [19]; however, there is no evidence for similar exonuclease activity in vertebrates.

miRNAs control the expression of the majority of human genes [20], and these molecules are involved in many physiological and pathological processes. The alteration of miRNA expression has been associated with numerous diseases, including neurodegenerative disorders, such as Huntington’s disease (HD). HD is the most common fatal polyglutamine (polyQ) disorder and results from the expansion of a CAG repeat in exon 1 of the huntingtin (*HTT*) gene. The precise mechanism of HD pathogenesis is not fully understood, but both the mutant protein (reviewed in [21]) and mutant transcript might be toxic to cells (reviewed in [22]). Of particular interest is the potential involvement of miRNA in the regulation of the *HTT* gene. The global deregulation of miRNAs in samples obtained from HD patients was demonstrated using Illumina massively parallel sequencing [23]. Most importantly, miRNA of varying lengths and/or sequences (isomiRs) were observed for the vast majority of miRNAs detected in two forebrain areas, the frontal cortex (FC) and striatum (ST), of both healthy individuals and HD patients [23].

In general, the miRNA heterogeneity observed in deep sequencing might have important functional implications. Most importantly, miRNAs with shifted 5′-ends have different seed sequences responsible for the recognition of a complementary sequence and the binding to mRNA. Therefore, it is assumed that heterogeneous 5′ isomiRs might regulate different targets [10,15,24,25]. Moreover, both 5′ and 3′ isomiRs might exhibit modified turnover properties [24,26] and altered strand selection within the RISC because strand selection is influenced by the extent of the 3′ overhang and the degree of pairing for any miRNA-miRNA* duplex [27,28].

An early evidence supporting the hypothesis of isomiRs functionality comes from an experiment that showed a difference in target cleavage between miR-142-5p and its variant, which contained two extra nucleotides at the 5′-end [29]. A putative functional role for isomiRs has been suggested in many reports because isomiRs actively associate with the RISC and translational machinery [24,30–32] (reviewed in [33]). This assumption was further supported by the observation that isomiRs exhibit differential expression across tissues and developmental stages [26,34,35]. Nevertheless, the real biological significance of isomiRs is not fully understood because few studies concerning isomiR regulation at the cellular level have been reported, and thus far, only variants of miR-133, miR-101, and miR-31 have been experimentally examined. Specifically, it was shown that 5′-isomiR-101, which is highly expressed in the brain, associates with AGO2 immunocomplexes and decreases the expression of five validated miR-101 targets but to a lesser degree than the canonical miR-101 [35]. Differential mRNA targeting was demonstrated in the case of two prevalent 5′ isomiRs of the key cardiac regulator miR-133a [31]. Three miR-31 isoforms that differed only slightly in their 5′- and/or 3′-end sequences were compared (namely, hsa-miR-31, ptr-miR-31, and mmu-miR-31), implicating isomiR-31s in the concordant and discordant regulation of six known target genes [36].

In this paper, we validated miRNA-mRNA interactions that might be involved in the regulation of the HTT transcript. Specifically, we experimentally assessed the validity of three predicted interactions and demonstrated that the canonical miR-137, miR-214, and miR-148a bind to the 3′UTR of the *HTT* gene. These results provide the first evidence that miR-137 and miR-148a regulate the expression of huntingtin and confirm that this regulation is also mediated by miR-214, as previously reported [37]. Moreover, using luciferase reporter assays, we investigated the regulation of huntingtin using select miRNA isoforms. We focused on 5′-end isomiRs with the shifted seed sequence that is the primary determinant of mRNA target recognition. Here, we showed that certain 5′-end isomiRs of miR-214 are functional for the downregulation of huntingtin expression.

## 2. Results and Discussion

### 2.1. Prediction of miRNA Interactions with the HTT Transcript

In a previous study, we predicted potential miRNA interactions with mRNAs derived from genes triggering hereditary neurological disorders known as trinucleotide repeat expansion diseases (TREDs), including Huntington’s disease (HD) [38]. The results of this in-depth *in silico* analysis prompted further research on the potential miRNA-mediated regulation of the HTT transcript in the context of the pathogenesis and therapy of HD. We compared different target prediction algorithms and verified our predictions using the available data gathered in various databases dedicated to miRNA target prediction (e.g., miRWalk database [39] and miRTarBase [40]). We selected interactions with miR-137, miR-214, and miR-148a for experimental verification. The deregulation of the expression of these miRNAs in HD patients or in cellular models of HD has been reported. Specifically, miR-137 was downregulated in the striatum of HD patients [23], while both miR-214 and miR-148a were upregulated in ST*Hdh^Q111^/Hdh^Q111^* cells [41]. Moreover, miR-137 is highly expressed in the nervous system, suggesting the involvement/potential role of this miRNA in the pathogenesis of HD. miR-137 has also been recently identified as a direct target of the repressor element-1 (RE-1) silencing transcription factor (REST) [42]. The second candidate, miR-214, has been positively verified in previous studies; miR-214, along with three other miRNAs (miRs 150, 146a, and 125b), downregulated the expression of huntingtin [37]. The same study also showed that these miRNAs affect the formation of mutHTT aggregates, the toxicity induced by mutHTT, and the expression of brain-derived neurotrophic factor (BDNF), thereby collectively contributing to HD pathogenesis.

**Figure 1:**
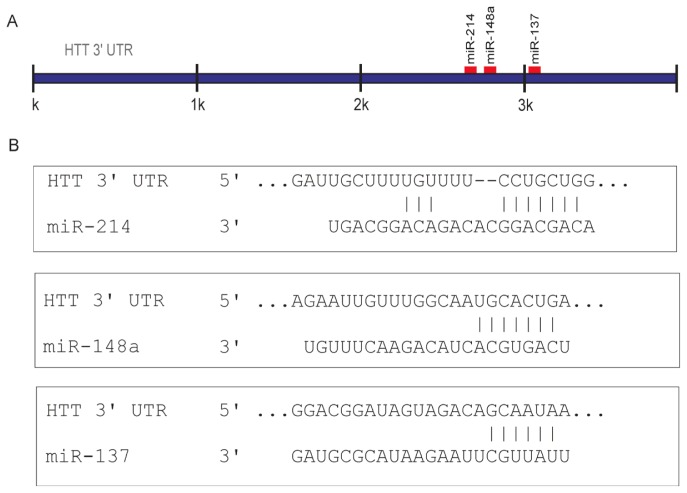
Graphical presentation of selected miRNA target site distribution in the 3′ untranslated region (3′UTR) of the huntingtin transcript. To predict miRNAs that potentially target the HTT 3′UTR, the TargetScanHuman algorithm (Release 6.2) [46] was used. (**A**) Regions of interaction for the miRNAs selected for experimental validation; (**B**) miRNA base pairing with an appropriate target sequence is schematically presented.

The candidate miRNAs (miRs 137, 214, and 148a) ranked high in the results generated by either algorithm based on conservation criteria, *i.e.*, Diana-micro T [43], miRanda [44], or PicTar [45]. However, our prediction was primarily based on the use of the TargetScanHuman algorithm (Release 6.2) [46]. According to TargetScan, a site for miR-137 is highly conserved among vertebrates, and sites for miRs 214 and 148a are poorly conserved among mammals or vertebrates. In addition, the miR-137 and miR-148a sites were 8mers (defined as exact matches to positions 2–8 of the mature miRNA, followed by an adenine), while the selected miR-214 site was a 7mer-m8 (an exact match to positions 2–8 of the mature miRNA). The positions of the miR-137, miR-214, and miR-148a binding sites in the 3′UTR of the huntingtin transcript and the base pairing of these miRNAs with target sequences are presented in Figure 1. The binding parameters of these miRNAs met the recommended bioinformatics criteria, and their experimental validation was of particular interest in the light of current knowledge of potential involvement of miRNAs in neurodegeneration and the entire competing endogenous RNA (ceRNA) activity network [47], which recently has been shown to be implicated in neurodegenerative diseases including HD [48,49].

### 2.2. Canonical miR-137, miR-214, and miR-148a Regulate the Expression of the HTT Gene

For the experimental validation of the predicted binding of the selected canonical miRNAs (miRs 137, 214, and 148a) to their target sites in the HTT 3′UTR, experiments using reporter constructs and luciferase assays were performed as described previously [50]. However, sequences carrying binding sites for the appropriate miRNAs were cloned into pmirGLO vector (Promega), which is considered optimal for miRNA-mRNA interaction studies. Constructs bearing single miRNA binding sites were generated and defined as wild-type reporters (WT). Constructs with mutations that disrupted native pairing within the binding region (5′ seed site) of the candidate miRNAs (MUT) and constructs that showed perfect complementarity (PM) to these sites were also generated to provide negative and positive controls, respectively (details in the Experimental section).

**Figure 2:**
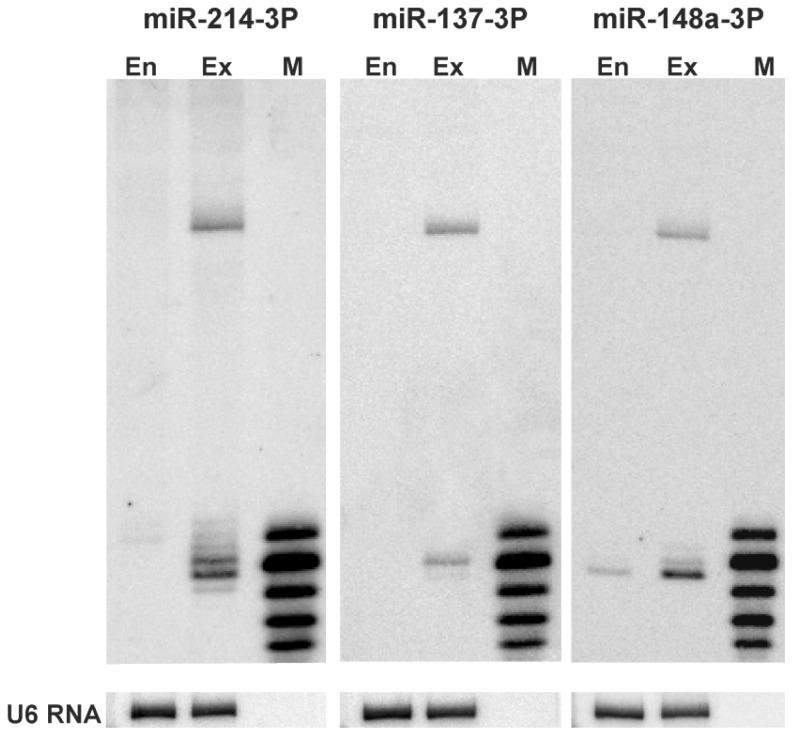
Endogenous expression and overexpression of miR-137, miR-214, and miR-148a in HEK293T cells. Northern blot detection of miRs 137, 214, and 148a in non-treated HEK293T cells and cells transfected with miRNA-coding plasmids (System Biosciences, Open Biosystems). M denotes the size marker, end-labeled 17, 19, 21, 23, and 25-nt oligoribonucleotides. En and Ex indicate the miRNA levels, endogenous and expressed from appropriate vectors, respectively. Hybridization to U6 RNA provides a loading control.

We transfected HEK293T cells with either reporter carrying potential miRNA binding sites. Four constructs were transfected into cells and tested in parallel. To determine whether the miRNAs of interest were expressed in the HEK293T cells, we performed northern blot analysis. The expression of miR-137 was not detected in the HEK293T cells. miR-214 and miR-148a were expressed at low and moderate levels (Figure 2), respectively, consistent with the available deep sequencing results. Therefore, our experimental system required miRNA overexpression, and we used miRNA-coding plasmid vectors (System Biosciences, Open Biosystems) for this purpose (compare endogenous miRNA levels and those expressed from vectors in Figure 2).

**Figure 3:**
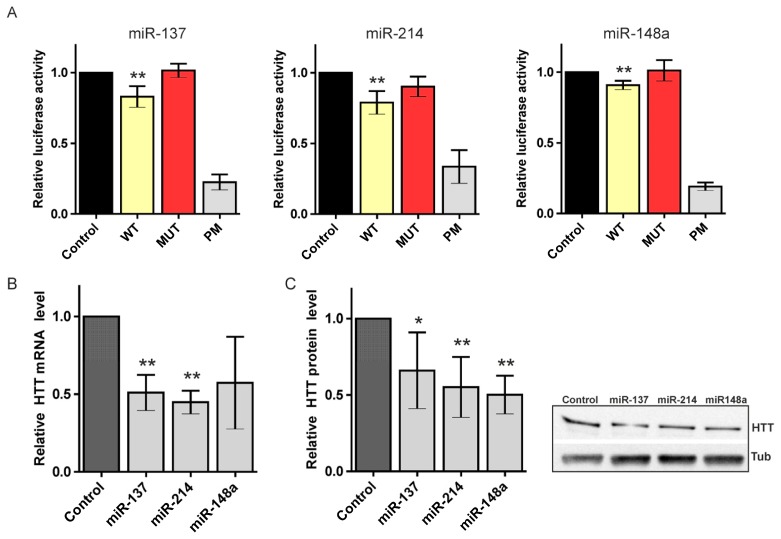
Regulation of the huntingtin (HTT) expression by canonical miRNAs. (**A**) Relative repression of the luciferase expression. Reporter constructs carrying a single binding site for miR-137, miR-214, and miR-148a were tested. For each luciferase. experiment, the miRNA activity on four constructs was measured in parallel: an empty pmirGlO vector (Control), a wild-type potential binding site for the appropriate miRNA (WT), a mutated binding site (MUT), and a site with full complementarity (PM). The firefly luciferase activity was normalized against *Renilla* luciferase activity. An average result from at least three independent experiments is shown (details in the text); (**B**) Relative HTT mRNA levels. Real-time PCR performed 48 h after transfection of HEK293T cells with miR-137, miR-214, and miR-148a. The bar graphs show the quantification of the HTT mRNA levels normalized to actin mRNAs based on data collected from three independent experiments; (**C**) Relative HTT protein levels. Western blot analysis of the cellular levels of HTT protein 72 h after transfection of HEK293T cells with miR-137, miR-214, and miR-148a. The bar graphs show the quantification of the protein levels detected in three western blot experiments. A representative blot is shown. The asterisks indicate statistical significance; a single asterisk at *p*-value < 0.05 and a double asterisk at *p*-value < 0.01.

In the luciferase assays, we obtained considerable repression of the luciferase expression after the transfection of reporter constructs for the three miRNAs tested (Figure 3A). Specifically, we observed a significant reduction in luciferase activity when reporter constructs bearing binding sites for miRs 137 and 214 were used (reductions to 83% and 79%, respectively) and a slightly weaker but reproducible and statistically significant suppression of the luciferase activity in the case of miR-148a (suppression to 87%). The luciferase activity for all of the MUT constructs showed efficient de-repression nearly equal to that in the control experiment; the positive controls (PMs) repressed luciferase at low levels, ranging from 17% to 33% (for miR-148a PM and miR-214 PM, respectively) of the empty reporter construct. These results verify the reliability of the experimental system used.

We also monitored huntingtin expression at the mRNA and protein levels following the transfection of HEK293T cells with miRNA-coding plasmids. Real-time PCR performed 48 h after transfection with miR-137, miR-214, or miR-148a showed a strong decrease in the HTT mRNA level (Figure 3B). Similarly, the HTT protein level was significantly reduced 72 h posttransfection in cells overexpressing any of the miRNAs (Figure 3C, Figure S1). This observation is consistent with the finding that miRNA binding reduces the cellular levels of targeted transcripts [51,52]. However, other studies have reported that no or minimal changes in the respective mRNA levels were observed or that these changes were only reported for certain targets [35]. Overall, miR-137, -214 and -148a were positively verified as negative regulators of the *HTT* gene. The lack of regulation of the huntingtin expression, demonstrated in both luciferase assays and western blotting, was observed for the other miRNA (miR-107) and shown for comparison as supplementary data (Figure S2). The strongest reduction in the luciferase activity and the greatest and second-greatest repression at the mRNA and protein levels were observed with miR-214. Thus, this study provides further support for the regulatory potential of miR-214, which was previously validated in a different experimental system [37]. Moreover, this study provides the first evidence of HTT regulation by miR-137 and miR-148a.

### 2.3. 5′-End Variants of miRNAs Are Functional and Might Regulate the Same Targets as Canonical miRNAs

Although many reports suggest isomiR functionality [24,30–32], there is still little research that address this issue experimentally. Specifically, one variant of miR-101 [35] and two isomiRs of miR-133 [31] and miR-31 [36] have been investigated. In these reports, the isomiRs were less effective than their canonical analogs [35] or exhibited differences in effectiveness depending on the regulated target [31].

**Figure 4:**
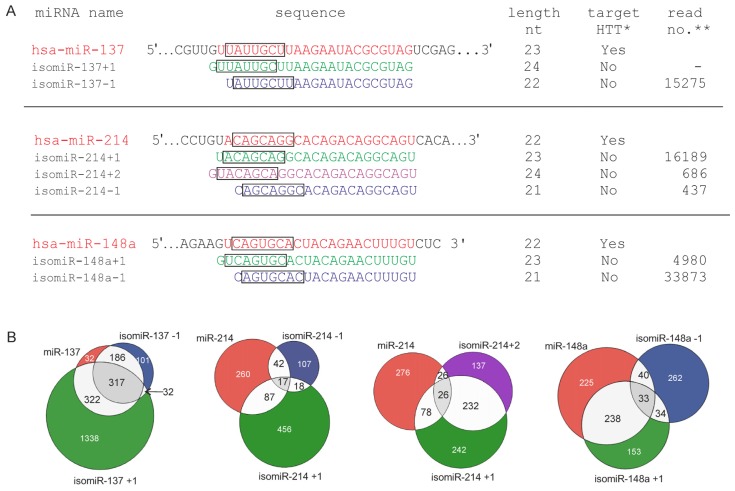
Graphical presentation of selected isomiR variants and their potential to target different genes. (**A**) Nucleotide sequences of miR-137, miR-214, miR-148a, and their isoforms. miRNA sequences are marked in red, and isomiR sequences are shown in blue, green, and violet for −1-, +1-, and +2-nt seed shifting, respectively. The miRNA seed sequences are labeled with black rectangles. Information on the miRNA lengths, as well as their potential for targeting the *HTT* gene and isomiR expression levels, is also provided. ^(^*^)^ Ability to interact with the HTT 3′UTR, as predicted by the TargetScanHuman algorithm (Release 6.2) for miRNAs and the TargetScan Custom (Release 5.2) for isomiRs [46], ^(^**^)^ isomiR read number according to the YM500 database [53]; (**B**) Venn diagrams showing the predicted miRNA targets for selected isomiRs. Potential targets for the 5′-end variants of miR-137, miR-214, and miR-148a were predicted using the TargetScan Custom algorithm (Release 5.2) and are shown as overlaps in the Venn diagrams. Targets for the canonical miRNAs are compared with the targets for the miRNAs with the shifted seed regions and are depicted in the same colors as in panel A. The numbers inside the circles denote the numbers of potential targets predicted for the appropriate miRNA variants.

Here, we determined whether the 5′-end variants of three miRNAs (5′-end isomiRs), namely miRs 137, 214 and 148a, might function in the same experimental system (*i.e.*, whether these miRs reduce the luciferase activity when appropriate reporter constructs are used). We designed and synthesized miRNA variants with seed sequences shifted by −1, +1, or +2 nt (Integrated DNA Technologies) (Figure 4A). We selected miRNA 5′ isoforms that are relatively highly represented in deep sequencing data because we considered sequence abundance a prerequisite for the functionality of these molecules. We based this selection on the sequencing data gathered in the YM 500 database [53] but we also evaluated the expression levels of isomiRs in other sources [32]. The only exception was isomiR-137+1, whose sequence is barely detectable using deep sequencing. This isomiR variant was added to the analysis to examine the same miRNA seed shifts for all isomiRs tested. Moreover, trimming variants that affect the 5′ end of miRNAs were reported to be abundant species, and the vast majority of these 5′ isomiRs affected a single nucleotide upstream of the reference miRNA [35]. A strong correlation between the expression of miRNAs and isomiRs was also observed [30].

According to the TargetScanHuman Custom (Release 5.2) [46] prediction, none of the selected isomiRs targeted HTT (Figure 4A); thus, we verified the targeting of these molecules experimentally. In addition, we assessed *in silico* how the overall number of genes targeted by the analyzed miRNAs and isomiRs might vary due to the change introduced into their seed regions. Potential targets for the 5′-end variants of miR-137, miR-214, and miR-148a were predicted using the TargetScan Custom 5.2 algorithm [46] and are shown in the Venn diagrams by overlaps (Figure 4B). Specifically, targets for the canonical miRNAs were compared with the targets of the miRNAs with seed regions shifted by −1, +1, and +2 nt. This analysis revealed that the number of predicted targets changed, but apart from unique targets, many genes were still predicted as targets for both miRNAs and isomiRs, confirming that isomiRs might share certain common mRNA targets but not all mRNA targets [36]. These results are also consistent with the suggestion that isomiRs function cooperatively to target common biological pathways [30]. However, distinct functions for miRs and isomiRs have also been suggested [31,35].

**Figure 5:**
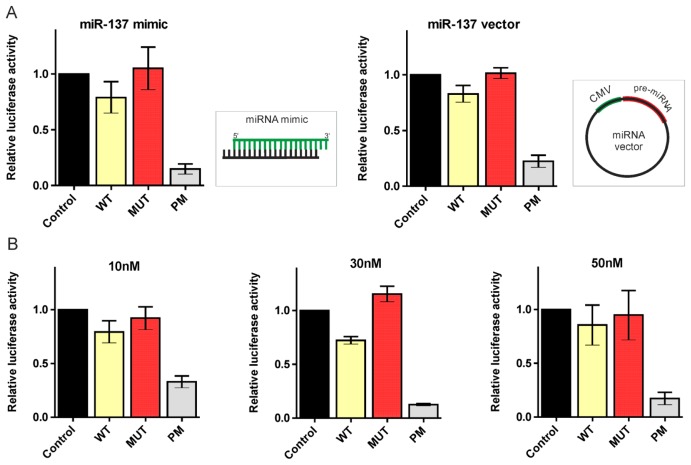
Correlation between the results of the luciferase experiments conducted with miR-137-coding plasmid and synthetic miR-137 mimics. (**A**) Relative repression of the luciferase expression. Reporter constructs carrying a single binding site for miR-137 were tested; miRNA activity on four constructs was measured in parallel (Control, WT, MUT, and PM), as described in Figure 3. Left—miRNA expression from the synthetic oligonucleotide (miR-137 mimic), right—miRNA overexpressed from the miR-137 vector. The firefly luciferase activity was normalized against *Renilla* luciferase activity. The standard errors are calculated from three independent experiments; (**B**) The relative repression of the luciferase expression resulted from the miRNA mimic activity. Four reporter constructs were tested (Control, WT, MUT, and PM) but with the addition of miR-137 mimic at different final concentrations, specifically 10, 30, and 50 nM, as denoted in the figure. The standard errors were calculated from one experiment performed in triplicate.

**Figure 6:**
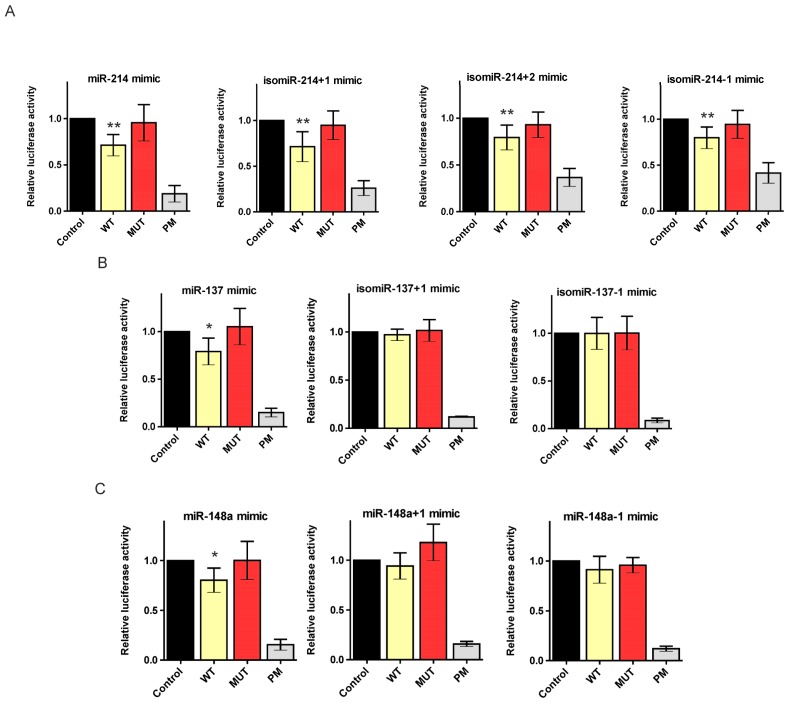
Regulation of the huntingtin expression by isomiRs. Relative repression of the luciferase expression for miR-214, miR-137, miR-148a, and their isomiRs (+1, +2, or −1). Reporter constructs carrying single binding sites for the appropriate miRNAs were tested, namely miR-137 (**A**), miR-214 (**B**), and miR-148a (**C**), as depicted in the figure. For each luciferase experiment, the miRNA activity on four constructs (Control, WT, MUT and PM) was measured in parallel, as described in Figures 3 and 5. The firefly luciferase activity was normalized against *Renilla* luciferase activity. The standard errors were calculated from three independent experiments. The asterisks indicate statistical significance; a single asterisk at *p*-value < 0.05 and a double asterisk at *p*-value < 0.01.

To validate the regulation of the HTT transcript by canonical miRNAs in a luciferase assay, we overexpressed the desired miRNAs from plasmid vectors. To study the interactions of the HTT transcript with isomiRs, appropriate isomiR sequences had to be introduced into cells as synthetic oligonucleotides. Thus, we transfected HEK293T cells with both the miR-137 mimic and miR-137 vector (System Biosciences) to determine whether these two experimental systems generate the same results (Figure 5A). Moreover, we examined miRNA mimic activities at different final concentrations (10, 30, and 50 nM) to determine the optimal concentration for these experiments (Figure 5B). A clear correlation between the results of the luciferase experiments with the miR-137-coding plasmid and the synthetic miR-137 mimic was observed; thus, we further investigated the functionality of our 5′-end isomiRs using appropriate miRNA mimics. In the luciferase assays, we obtained considerable and significant repression of the luciferase expression after the transfection of the reporter constructs and all three miR-214 isomiR mimics, namely, isomiR-214+1, isomiR-214+2, and isomiR-214-1 (luciferase repression equal to 71%, 80%, and 79%, respectively). Moreover, this reduction in the luciferase activity was comparable to the reduction induced by the canonical miR-214 mimic (71%) (Figure 6A). In contrast, the luciferase activity was not reduced when miR-137 isomiRs were used, in the case of neither isomiR-137+1 nor isomiR-137-1, compared with the considerable repression observed using the canonical miR-137 mimic (79%) (Figure 6B). Similarly, in the case of isomiR-148a+1 and isomiR-148a-1, the activity of luciferase was slightly reduced (9% and 6%, respectively), while the reduction obtained for the canonical miR-148a mimic was much stronger (80%) (Figure 6C). The observed difference in the functionality of the analyzed isomiRs raises the question when miRNA-mRNA pairing conforms to strict rules and when some flexibility in the miRNA seed region is permitted, and which additional mechanisms other than the base paring of the seed region might affect target genes repression by isomiRs.

Several factors influence the recognition of a target site by miRNA, e.g., the sequence composition of the 3′-UTR [54], the immediate environment of the putative target site [55], and the structural accessibility of the target site [2,56]. Moreover, endogenous natural antisense transcripts transcribed from the opposite strand of a protein-coding gene or a non-protein coding gene [34] and the RNA-binding proteins [57] could directly bind to mRNA, thereby masking the miRNA binding site of a target gene and preventing the inhibitory effects of the miRNA on target gene translation. These factors, however, are of importance to canonical miRNA binding. Here, we examined several 5′ isomiRs of slightly different lengths that previously demonstrated canonical miRNA targeting. Therefore, the structural features and genomic context of these molecules did not significantly differ between the canonical miRNAs and their isomiRs or between the isomiRs themselves.

A distinct feature of the functional isomiR-214 variants and the two other isomiRs examined in this study was the fact that miR-214 is a 7mer with compensatory base pairing at the 3′ end (see Figure 1). Although canonical miRNA-target specificity is primarily triggered by complementarity within the seed region, non-canonical interactions depend also on 3′ compensatory sites [2,58], which might be important for miR-214 and its variants. The miRNA/isomiR length was also suggested as a factor that might affect functionality. In a study of isomiRs, the analysis of two miR-133a mimics (22/23 nt) was performed, followed by the analysis of two other variants that represented the respective other length for each miR-133a variant. However, the luciferase repression did not depend on mimic length within this range [31]. Therefore, alterations to the 3′ end of the miR-133a mimic did not affect the level of mRNA repression, suggesting that the 3′ end is not essential for efficient target binding in this case. Another important factor that might account for the disparate functioning of isomiRs is differential binding capacity with the Argonaute complex (affinity of a given miRNA to AGO). Previous studies have shown that some miRNA variants were differentially loaded onto AGOs, and the 5′-end nucleotide of small RNA was critical for its interaction with AGO proteins [12,59–61]. However, miR-101 was more efficiently loaded into the RISC than its isomiR [35], and the 5′-end nucleotide of isomiR-31s was not a rigorous criterion for AGO complex loading [36]. In this study, in the case of the most effective miRNA, namely miR-214, all variants were functional regardless of the different nucleotides at their 5′ end (Figure 2). Small changes in the miRNA sequence profoundly affected the functional asymmetry of the miRNA duplex, altering which strand of a miRNA duplex functions in mRNA silencing [18]. Therefore, it cannot be ruled out that, in the case of the nonfunctional isomiRs of miR-137 and miR-148a, the passenger strands were incorporated into the RISC and did not target their binding sites.

## 3. Experimental Section

### 3.1. Cell Culture

HEK293T cells were obtained from the American Type Culture Collection (ATCC) and grown in Dulbecco’s Modified Eagle’s Medium (DMEM, Lonza, Wakersville, MD, USA) supplemented with 8% fetal bovine serum (FBS) (Sigma-Aldrich, St. Louis, MO, USA), 2 mM l-glutamine, and an antibiotic-antimycotic solution (Sigma-Aldrich, St. Louis, MO, USA) at 37 °C in a humidified atmosphere of 5% CO_2_. At 24 h prior to transfection, the HEK293T cells were plated in 12-well or 6-well dishes in DMEM growing medium and harvested 24, 48, and 72 h post-transfection for the luciferase assay, real-time PCR, and western blot analyses, respectively.

### 3.2. Plasmid Constructs and Synthetic miRNA Oligonucleotides

To generate reporter constructs bearing miRNA-binding sites, the pmirGLO Dual-Luciferase miRNA Target Expression Vector was used (Promega, Madison, WI, USA). This vector is based on Promega dual-luciferase technology, with firefly luciferase (*luc2*) as the primary reporter for monitoring mRNA regulation and *Renilla* luciferase (*hRluc-neo*) as a control reporter for normalization and selection. Specific oligonucleotides with *Dra*I and *Xba*I ends containing single binding sites for the analyzed miRNA (HTT b.s. for miRs 214, 137, and 148a) were synthesized (IBB Warsaw). The appropriate oligos were annealed by boiling and gradual cooling and subsequently phosphorylated and cloned into the pmirGLO vector, previously digested with *Dra*I (Fermentas, St.-Leon-Rot, Germany) and *Xba*I (Fermentas, St.-Leon-Rot, Germany) restriction enzymes, downstream of the *luc2* gene. For all miRNAs, three types of constructs were prepared, namely wild type (WT), carrying mutations (MUT) and perfect match (PM) constructs (for sequences refer to Table S1), which all have 10-nucleotide flanking sequences, as described previously [50].

For miRNA overexpression, commercial plasmid constructs expressing miRNA precursors (pri-miR-148a (Open Biosystems, Huntsville, AL, USA), pri-miR-137, or pri-miR-214 (System Biosciences, Mountain View, CA, USA)) were used. These plasmids contain pri-miRNA sequences in their natural genome context to ensure biologically relevant interactions with the endogenous processing machinery.

Synthetic miRNA mimics (miR-137, miR-214, and miR-148a mimics) and their length variants were chemically synthesized (Integrated DNA Technologies). The following modifications were introduced: (1) 2′-*O*-methyl modification on positions 1 and 2 and a two-nucleotide UU overhang on the 3′ end of the miRNA mimic sense strand, (2) 5′ phosphorylation and a two-nucleotide overhang based on nucleotide types found in natural pre-miRNAs on the 3′ end of the miRNA mimic antisense strand. All sequences are presented as supplementary data (Table S2).

### 3.3. Cell Transfection

HEK293T cells were transfected using Lipofectamine 2000 (Invitrogen, Carlsbad, CA, USA) according to the manufacturer’s protocols. For luciferase assays, the cells were transfected in 12-well plates at ~80% confluence. For each transfection experiment, 200 ng of the appropriate reporter construct and either 250 ng of the appropriate miRNA-coding vector or 30 nM of miRNA mimic were used. The cells were harvested 24 h after transfection and assayed for luciferase activity. For miRNA overexpression required for real-time PCR and western blot analyses, the cells were grown to 80% and 60% confluence, respectively, transfected in 6-well plates with 1 μg/mL pri-miRNA plasmid vectors, and harvested at 48 and 72 h, respectively.

### 3.4. Luciferase Reporter Assay

After harvesting, the cells were lysed in a passive lysis buffer (Promega, Madison, WI, USA). The luciferase activity was measured using a Dual-Luciferase Reporter Assay System (Promega, Madison, WI, USA) according to the manufacturer’s instructions with a Centro LB 960 luminometer (Berthold Technologies, Oak Ridge, TN, USA).

### 3.5. RNA Isolation and Real-Time PCR

Total RNA from HEK293T cells was isolated using TRI Reagent (MRC, Inc., BioShop, Cincinnati, OH, USA) according to the manufacturer’s instructions. The RNA concentration was estimated using a NanoDrop spectrophotometer. cDNA was obtained from 500 ng of total RNA using Superscript III (Life Technologies, Carlsbad, CA, USA) and random hexamer primers (Promega, Madison, WI, USA). For subsequent quantitative real-time analyses, 50 ng of cDNA was used. Real Time PCR was performed on a LightCycler 480 II system (Roche Diagnostics, Mannheim, Germany) using TaqMan Gene Expression Assays and TaqMan Universal Master Mix II (Applied Biosystems, Foster City, CA, USA). The results obtained for the assessment of huntingtin mRNA levels were normalized to the levels of actin mRNA.

### 3.6. Northern Blotting

High-resolution northern blotting was performed as previously described [62,63]. Briefly, 25 μg of total RNA was extracted from HEK293T cells and resolved on a 12% denaturing polyacrylamide gel in 0.5× TBE. The RNA was transferred to a GeneScreen Plus hybridization membrane (PerkinElmer, Spokane, WA, USA) using semi-dry electroblotting (Sigma-Aldrich, St. Louis, MO, USA), immobilized by subsequent UV irradiation (120 mJ/cm^2^) (UVP), and baked in an oven at 80 °C for 30 min. The membranes were probed with specific DNA oligonucleotides (Table S3) complementary to the annotated human miRNAs miR-137-3P, miR-214-3P, and miR-148a-3P (miRBase). The probes were labeled with [γ^32^P] ATP (5000 Ci/mmol; Hartmann Analytics, Braunschweig, Germany) using USB OptiKinase (Affymetrix, Cleveland, OH, USA). The hybridizations were performed at 37 °C overnight in a PerfectHyb buffer (Sigma-Aldrich, St. Louis, MO, USA). The marker lanes contained a mixture of radiolabeled RNA oligonucleotides (17-, 19-, 21-, 23-, and 25-nt in length). Hybridizations to U6 RNA provided loading controls. Radioactive signals were quantified by phosphorimaging (Multi Gauge v3.0; Fujifilm).

### 3.7. Western Blotting

A total of 15 μg of protein was diluted in sample buffer containing 2-mercaptoethanol, denatured for 5 min, and separated on 3%–8% gradient Tris-Acetate gels (Invitrogen, Carlsbad, CA, USA) in XT Tricine Buffer (BioRad, Hercules, CA, USA). After electrophoresis, the proteins were electrotransferred onto a nitrocellulose membrane (Sigma, St. Louis, MO, USA). All immunodetection steps were performed on a SNAPid (Millipore, Billerica, MA, USA) in PBS buffer containing 0.25% nonfat milk and 0.1% Tween 20, and the membranes were washed in PBS/Tween. For huntingtin and tubulin detection, the blots were probed with the primary anti-huntingtin (1:500, Millipore, Billerica, MA, USA) and anti-alpha-tubulin (1:5000, Covance, Emeryville, CA, USA) antibodies, respectively, and subsequently probed with HRP-conjugated secondary antibodies (1:500, Sigma, St. Louis, MO, USA). The immunoreaction was detected using Western Bright Quantum (Advansta, CA, USA). The protein amounts were quantified using GelPro 3.1 software (Media Cybernetics, Bethesda, MD, USA).

### 3.8. Statistical Analysis

All experiments were repeated at least three times. Graphs were generated using GraphPad Prism 5 (GraphPad Software). The figures for the luciferase assays were generated after averaging the results from the repeat experiments for a particular construct. The values for error bars (mean with SD) and the statistical significance were calculated using GraphPad Prism 5. The statistical significance of the luciferase reduction in the case of transfection with constructs carrying miRNA-binding sites was assessed using a one-sample *t*-test with a hypothetical value of 1 assigned to cells transfected with a control empty vector. *p*-values < 0.05 (two-tailed) were considered significant.

## 4. Conclusions

This study presents new evidence that *HTT* gene expression is regulated by miRNAs and, most importantly, demonstrates that certain isomiRs are functional and regulate the same target as canonical miRNAs.

IsomiRs are commonly reported in deep-sequencing studies and have been described in all studied organisms and tissues. The existence of miRNA variants might contribute considerably to the complexity of target regulation by miRNAs and strongly increase the regulatory potential of these molecules. The presence of isomiRs could have far-reaching implications for miRNA therapeutic applications; it must be taken into account in various diagnostic tests as well as in the design of miRNA mimics or anti-miRs as therapeutic agents. Therefore, of particular importance is to identify factors that determine the biological relevance of isomiRs.

## Supplementary Information
