# Supplementary material for: Regulation of Huntingtin Gene Expression by miRNA-137, -214, -148a, and Their Respective isomiRs
Source: Int J Mol Sci. 2013 Aug 19;14(8):16999–7016. doi: 10.3390/ijms140816999 (PMC3759948; doi:10.3390/ijms140816999)
Supplement: Supplementary file 1 [file ijms-14-16999-s001.pdf]

Supplementary Information

Table S1. The sequences of miRNA binding sites cloned in reporter constructs.

miRNA	Construct strand	Sequence 5'-3'
miR-137	WT 1	AAAGTGGAGCTCGGGACGGATAGTAGACAGCAATAACTCGGTGTGTT
	WT 2	CTAGAACACACCGAGTTATTGCTGTCTACTATCCGTCCCGAGCTCCACTTT
	MUT 1	AAAGTGGAGCTCGGGACGGATAGTAGACATCGACAACTCGGTGTGTT
	MUT 2	CTAGAACACACCGAGTTGTCGATGTCTACTATCCGTCCCGAGCTCCACTTT
	PM 1	AAAGTGGAGCTCGCTACGCGTATTCTTAAGCAATAACTCGGTGTGTT
	PM 2	CTAGAACACACCGAGTTATTGCTTAAGAATACGCGTAGCGAGCTCCACTTT
miR-214	WT 1	AAACTGTGGGGAGATTGCTTTTGTTTTCCTGCTGGTAATATCGGGTT
	WT 2	CTAGACCCGATATTACCAGCAGGAAAACAAAAGCAATCTCCCCACAGTTT
	MUT 1	AAACTGTGGGGAGATTGCTTTTGTTTTACCTCCGGTAATATCGGGTT
	MUT 2	CTAGACCCGATATTACCGGAGGTAAAACAAAAGCAATCTCCCCACAGTTT
	PM 1	AAACTGTGGGGAGAAACTGCCTGTCTGTGCCTGCTGTATATCGGGTT
	PM 2	CTAGACCCGATATACAGCAGGCACAGACAGGCAGTTTCTCCCCACAGTTT
miR-148a	WT 1	AAATGAAACCAGGGTAGAATTGTTTGGCAATGCACTGAAGCGTGTT
	WT 2	CTAGAACACGCTTCAGTGCATTGCCAAACAATTCTACCCTGGTTTCATTT
	MUT 1	AAATGAAACCAGGGTAGAATTTGGCAAAGGAACGAAGCGTGTT
	MUT 2	CTAGAACACGCTTCGTTCCTTTGCCAAATTCTACCCTGGTTTCATTT
	PM 1	AAATGAAACCAGGACAAAGTTCTGTAGTGCACTGATGAAGCGTGTT
	PM 2	CTAGAACACGCTTCATCAGTGCACTACAGAACTTTGTCCTGGTTTCATTT

Table S2. Oligodeoxynucleotide sequences used as miRNA mimics.

Mimic name	Strand	Sequence 5'-3'
miR-137	antisense	UUAUUGCUUAAGAAUACGCGUAGUC
	sense	CUACGCGUAUUCUUAAGCAAUAAUU
isomiR-137+1	antisense	GUUAUUGCUUAAGAAUACGCGUAG
	sense	ACGCGUAUUCUUAAGCAAUAACUU
isomiR-137-1	antisense	UAUUGCUUAAGAAUACGCGUAGUC
	sense	CUACGCGUAUUCUUAAGCAAUAUU
miR-214	antisense	ACAGCAGGCACAGACAGGCAGUCA
	sense	ACUGCCUGUCUGUGCCUGCUGUUU
isomiR-214+1	antisense	UACAGCAGGCACAGACAGGCAGUCA
	sense	ACUGCCUGUCUGUGCCUGCUGUAUU
isomiR-214-1	antisense	CAGCAGGCACAGACAGGCAGUCA
	sense	ACUGCCUGUCUGUGCCUGCUGUU
isomiR-214+2	antisense	GUACAGCAGGCACAGACAGGCAGUCA
	sense	ACUGCCUGUCUGUGCCUGCUGUACUU
miR-148a	antisense	UCAGUGCACUACAGAACUUUGUCU
	sense	ACAAAGUUCUGUAGUGCACUGAUU
isomiR-148a+1	antisense	GUCAGUGCACUACAGAACUUUGUCU
	sense	ACAAAGUUCUGUAGUGCACUGACUU
isomiR-148a-1	antisense	CAGUGCACUACAGAACUUUGUCU
	sense	ACAAAGUUCUGUAGUGCACUGUU

Table S3. Oligodeoxynucleotide sequences used as northern blot probes.

Detected sequence	Probe sequence
miR-137-3P	CTACGCGTATTCTTAAGCAATAA
miR-214-3P	ACTGCCTGTCTGTGCCTGCTGT
miR-148a-3P	ACAAAGTTCTGTAGTGCACTGA

Figure S1. Reduction in the huntingtin protein levels after treatment with canonical miRNAs.

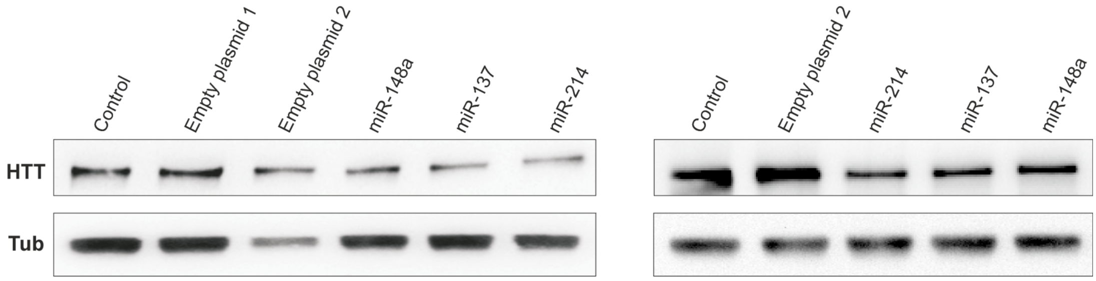

Figure S2. Lack of regulation of the huntingtin expression by miR-107.

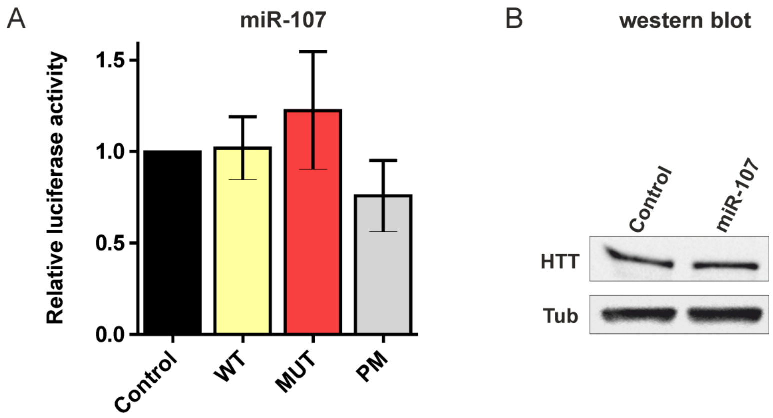

© 2013 by the authors; licensee MDPI, Basel, Switzerland. This article is an open access article distributed under the terms and conditions of the Creative Commons Attribution license (http://creativecommons.org/licenses/by/3.0/).
